# Orthopedic implant-associated infections caused by *Cutibacterium* spp. – A remaining diagnostic challenge

**DOI:** 10.1371/journal.pone.0202639

**Published:** 2018-08-20

**Authors:** Nora Renz, Stasa Mudrovcic, Carsten Perka, Andrej Trampuz

**Affiliations:** 1 Charité –Universitätsmedizin Berlin, corporate member of Freie Universität Berlin, Humboldt-Universität zu Berlin, and Berlin Institute of Health, Center for Musculoskeletal Surgery (CMSC), Berlin, Germany; 2 Berlin-Brandenburg Center for Regenerative Therapies (BCRT), Berlin, Germany; University of Ulster, UNITED KINGDOM

## Abstract

**Background:**

The definition criteria and clinical characteristics of implant-associated infection (IAI) caused by *Cutibacterium* (formerly *Propionibacterium*) spp. are poorly known. We analyzed microbiologically proven *Cutibacterium* orthopedic IAI in a prospective cohort.

**Methods:**

Patients with periprosthetic joint infections (PJI) and fixation device–associated infections (FDAI) caused by *Cutibacterium* spp. were prospectively included. IAI was defined by significant growth of *Cutibacterium* spp. and presence of at least one non-microbiological criterion for infection. The McNemar’s chi-squared or binomial test was used to compare the performance of diagnostic tests.

**Results:**

Of 121 patients with *Cutibacterium* IAI, 62 patients (51%) had PJI and 59 (49%) had FDAI. 109 infections (90%) were caused by *C*. *acnes* and 12 (10%) by *C*. *avidum*. The median time from implantation until diagnosis of infection was 15.7 months (interquartile range, 5–46.5 months). Clinical local signs were present in 30 patients (28%) and radiological implant loosening in 64 patients (63%). Culture sensitivity of sonication fluid was 84%, of peri-implant tissue 84% and of synovial or peri-implant fluid 56% after 14 days of incubation.

**Conclusion:**

*Cutibacterium* IAI was diagnosed late in the disease course and presented with subtle signs. Prolonged culture incubation and implant sonication improved the poor performance of conventional microbiological tests. Due to lack of reliable diagnostic tests, *Cutibacterium* remains difficult to detect making the diagnosis challenging.

## Introduction

Bacteria belonging to the genus *Propionibacterium* are gram-positive, aerotolerant anaerobic rods that reside primarily in pilosebaceous follicles and are part of the normal skin microbiome. Based on species habitats, genomic topology, DNA G+C content and peptidoglycan composition, *Propionibacterium acnes*, *P*. *avidum*, *P*. *granulosum* and *P*. *humerusii* were recently proposed to be reclassified to the novel genus *Cutibacterium* [1]. In addition to the skin, *Cutibacterium* can be found in other parts of the body such as the oral cavity, gastrointestinal and genitourinary tract [2, 3], where they usually exist as non-pathogenic commensals. In the presence of an implant, *Cutibacteria* are increasingly recognized as the causative pathogen of low-grade infections affecting cardiovascular devices [4], breast implants [5], neurosurgical shunts [6], ocular implants [7], internal fracture fixation devices [8], spinal hardware [9, 10] and joint prostheses [11]. They claimed attention particularly in infections after shoulder arthroplasty [12–16], spine surgery [9, 10] and craniotomy [17].

As life expectancy is rising and novel technologies are being developed, the number of implanted devices is steadily increasing. With diagnostic techniques designed for improved diagnosis of implant-associated infections (IAI) and the use of better definition criteria for infection, *Cutibacterium* is increasingly recognized as true pathogen rather than contamination. These methods include prolonged incubation of microbiological specimens [2], application of novel techniques for biofilm detection, such as sonication of explanted materials [18] and implementation of molecular assays [19–21].

The reported frequency, type, treatment and outcome of IAIs caused by *Cutibacterium* spp. vary widely between countries, institutions and medical specialties, indicating that many challenges are unresolved, including the definition, detection and interpretation of this pathogen. Detection of *Cutibacterium* in culture or non-culture assays can be both, overestimated (i.e. misinterpretation of contaminant as pathogen) or underestimated (i.e. misinterpretation of pathogen as contaminant) [3, 22, 23]. Aiming at better understanding the pathogenetic role of *Cutibacterium* in orthopedic IAI, we conducted a prospective study to analyze the diagnostic, clinical and treatment characteristics using standardized definition criteria and an uniform diagnostic algorithm. We hypothesized, that sensitivity of diagnostic tests would be low for orthopedic IAI caused by *Cutibacterium* spp., rendering these infections difficult to diagnose.

## Patients and methods

### Study design

This prospective cohort study was conducted in a tertiary healthcare center, providing advanced specialty care to a population of 4 million inhabitants. Patient recruitment, data collection and follow-up evaluation were performed within the institutional implant infection cohort project. The study protocol was reviewed and approved by the institutional ethics committee (Ethikkommission der Charité –Universitätsmedizin Berlin) and was performed in accordance with the Declaration of Helsinki. The need for informed consent was waived.

### Study population

From January 2012 through March 2018 consecutive episodes of IAI caused by *Cutibacterium* spp. were included and classified as periprosthetic joint infections (PJI) and fixation device–associated infections (FDAI). Each episode was evaluated by an orthopedic surgeon and infectious diseases specialist according to predefined criteria (see below). Mixed infections including high-virulent microorganism (such as *Staphylococcus aureus*, *Escherichia coli* or streptococci) were excluded, whereas co-infections with low-virulent microorganisms were included. The patients were treated according to previously published treatment recommendations [24].

### Definitions

IAI was diagnosed when growth of *Cutibacterium* spp. in at least two intraoperative peri-implant tissue samples or sonication fluid of the removed implant (>50 CFU/ml) [18] was documented. If it only grew in synovial fluid, at least one of the following additional criteria had to be present to confirm infection [25, 26]: (i) macroscopic purulence around the implant as determined by the surgeon, (ii) presence of a sinus tract communicating with the implant, (iii) implant on view, (iv) acute inflammation in intraoperative sampled peri-implant-tissue as described by the histopathological report [27, 28] or (v) synovial fluid with >2000 leukocytes/μl or >70% granulocytes in case of PJI. Time to infection diagnosis was defined as the interval from implantation of the prosthesis or fixation device (or last surgical revision of implant) to the diagnosis of infection. IAIs were categorized according to the time of manifestation in early (<3 months after surgery), delayed (3–24 months after surgery) and late (>24 months after surgery) infections [24].

### Data and strain collection

Hospital charts were reviewed with a standardized case-report form to retrieve demographic, clinical, and laboratory data. The following data was extracted: age, sex, implant type, date of primary implantation, date of diagnosis, previous revision surgery, clinical signs and symptoms, systemic inflammatory biomarkers, microbiology (including antimicrobial susceptibility testing), histopathology, leukocyte count in synovial fluid (if available), antimicrobial and surgical therapy. The radiological images were assessed at time of infection diagnosis for signs of loosening, dislocation or heterotopic ossifications for joint prosthesis or insufficient bone consolidation, defined as either delayed union (at 4–6 months) or non-union (at >6 months) for fracture fixation devices [29]. *Cutibacterium* strains were collected and stored in the biobank at -80°C for further microbiological testing.

### Diagnostic tests

Joint aspiration was performed by an orthopedic surgeon according to standardized aseptic technique. After skin preparation with povidone iodine-alcohol, synovial fluid was aseptically collected using a sterile 18-gauge needle. If the joint was aspirated preoperatively in the outpatient department, a small skin incision was done before introducing the syringe. If no synovial fluid was obtained, the needle was repositioned without withdrawing it through the skin; no fluid was injected into the joint cavity. in case of intraoperative sampling during revision surgery, synovial fluid was aspirated before opening the joint capsule. For determination of leukocyte count and percentage of granulocytes, one ml of synovial fluid was transferred into a vial containing ethylenediaminetetraacetic acid (EDTA). In addition, one ml of synovial fluid was inoculated into a pediatric blood culture bottle (BacTec PedsPlus/F, Beckton Dickinson and Co., Shannon, County Clare, Ireland) and incubated at 36 ± 1°C for 14 days or until a positive growth was signaled, 0.1 ml of synovial fluid was inoculated on aerobic and anaerobic sheep blood agar plates (bioMérieux, Marcy L’Etoile, France) and incubated 7 days aerobically at 37°C with 5% CO2 and 14 days anaerobically at 37°C. The. remaining fluid was inoculated in thioglycolate broth (Becton–Dickinson and Company, USA) for enrichment. In addition, peri-implant fluid and 3 to 5 periprosthetic tissue samples were collected intraoperatively from the implant-bone or cement-bone interface for microbiological and histopathological analysis, if revision surgery was performed. Tissue samples were homogenized and inoculated on aerobic and anaerobic blood agar plates and inoculated in thioglycolate broth, as described above for synovial fluid. The retrieved prosthetic components were sent for sonication and processed within 6 h of removal, as previously described [30]. In brief, after adding normal saline covering most of the implant, the sonication box was vortexed for 30 s, sonicated for 1 min at 40 kHz (BactoSonic, Bandelin electronic, Berlin, Germany) and again vortexed for 30 s. The resulting sonication fluid was plated in aliquots of 0.1 ml onto aerobic and anaerobic sheep blood agar plates and 1 ml was inoculated in thioglycolate broth. Cultures were incubated at 37°C for 14 days and inspected daily for microbial growth. Microorganisms on plates were enumerated as the number of colony-forming unit (CFU)/ml sonication fluid. The colonies of each microorganism morphology were identified by standard microbiological methods using automated system VITEK 2 (bioMérieux, Marcy L’Etoile, France). Susceptibility testing was performed using gradient-strip test (E-test) by the hospital microbiology laboratory (Labor Berlin—Charité Vivantes GmbH).

### Statistical analysis

Categorical variables were compared using the Fisher's exact test, for comparison of continuous variables the Mann-Whitney U test was applied. A two-sided p-value of < 0.05 was considered significant. For statistical analyses and graphics the software Prism (version 7.03; GraphPad, La Jolla, CA, USA) was used.

## Results

### Patient characteristics

Fourty-eight patients were excluded because of non-significant growth of *Cutibacterium* spp. (i.e. single tissue positive or < 50 CFU/ml in sonication) and 10 patients were excluded because of co-infection with a highly virulent pathogen. Of the remaining 121 patients with orthopedic IAI caused by *Cutibacterium* spp., 62 patients (51%) had PJI (including 30 hip, 19 shoulder, 12 knee and one elbow prosthesis) and 59 patients (49%) had FDAI (affecting 27 spinal hardware devices, 20 plates, 5 anchorages after rotator cuff reparation, 4 intramedullary nails, 2 fixation devices for cruciate ligament graft and one dynamic hip screw) (Table 1). Patients with PJI were older than those with FDAI (71 years vs. 55 years, p < 0.001) and the infection more often involved the lower extremity (68% vs. 27%, p < 0.001). Revision surgery at the index implant was performed in 35 patients (29%), more commonly reported in PJI than in FDAI (38% vs. 20%, p = 0.044).

**Table 1:** Characteristics of 121 patients with orthopedic implant-associated infections, including 62 with periprosthetic joint infections (PJI) and 59 with fixation device-associated infection (FDAI).

Characteristic		All patients (n = 121)		Patients with PJI^a^ (n = 62)		Patients with FDAI^b^ (n = 59)		P value	
Median age in years		66 (IQR, 52–75)		71 (IQR, 62–76)		55 (IQR, 47–71)		< 0.001*	
Sex, male		82 (68%)		39 (63%)		43 (73%)		0.330^#^	
Anatomic location of implant									
Lower extremity		58 (48%)		42 (68%)		16 (27%)		< 0.001^#^	
Upper extremity		36 (30%)		20 (32%)		16 (27%)		0.557^#^	
Spine		27 (22%)		-		27 (46%)		-	
No. previous revisions on index implant									
None		84/119 (71%)		37/60 (62%)		47 (80%)		-	
≥1 interventions		35/119 (29%)		23/60 (38%)		12 (20%)		0.044^#^	

**NOTE**. Data are no. (%) of patients, if not indicated otherwise. P values were calculated between the PJI group and the FDAI group using Mann-Whitney U test (*) or Fisher’s exact test (^#^). IQR, interquartile range.

^a^ Including 30 hip, 19 shoulder, 12 knee and one elbow prosthesis.

^b^ Including 27 spinal hardware devices, 25 fracture-fixation devices (12 humerus, 5 tibia, 4 femur, 4 clavicle), 5 anchorages after rotator cuff reparation and 2 fixation devices for cruciate ligament graft.

### Infection characteristics

Clinical, laboratory and radiologic features of IAI are summarized in Table 2. The median time from implantation to onset of infection was 15.7 months (IQR, 5–46.5 months). The onset of infection in FDAI was earlier than in PJI (10.0 months vs. 33.8 months, p < 0.001). This difference was also reflected by the low proportion of early infections (7%) in the PJI group (4 of 60 patients), compared to 29% in the FDAI group (16 of 55 patients, p = 0.003). Persistent or increasing pain at joint site was the most frequent clinical symptom reported in 86 patients (80%), followed by local signs of inflammation in 30 patients (28%) and sinus tract in 9 patients (8%), whereas fever was documented in only one patient (1%). Median C-reactive protein and white blood cell count were in the normal range in both groups. Radiological loosening was reported in 64 patients (63%), heterotopic ossifications were described in x-ray in 16 of 53 patients (30%) with PJI.

**Table 2:** Infection characteristics of 121 orthopedic implant-associated infections, including 62 with periprosthetic joint infections (PJI) and 59 with fixation device-associated infection (FDAI).

Characteristic	All patients (n = 121)	Patients with PJI (n = 62)	Patients with FDAI (n = 59)	P value
Median time from implantation to onset of infection in months	15.7 (IQR, 5–46.5)	33.8 (IQR, 8.3–58.8)	10.0 (IQR, 2–23.3)	< 0.001*
Type of infection according to onset of infection after implantation				
Early (<3 months)	20/115 (17%)	4/60 (7%)	16/55 (29%)	0.003^#^
Delayed (3–24 months)	49/115 (43%)	22/60 (37%)	27/55 (49%)	0.192^#^
Late (>24 months)	46/115 (40%)	34/60 (57%)	12/55 (22%)	< 0.001^#^
Clinical findings				
Persistent or increasing pain at joint site	86/103 (80%)	42/55 (76%)	44/48 (92%)	0.061^#^
Local signs of inflammation^a^	30/108 (28%)	14/55 (25%)	16/53 (30%)	0.669^#^
Sinus tract	9/108 (8%)	6/55 (11%)	2/29 (7%)	0.708^#^
Fever (>38°C) at admission	1 (1%)	0 (0%)	1 (2%)	0.487^#^
Radiological findings				
Migration or loosening of the implant	64/101 (63%)	36/53 (68%)	28/48 (58%)	0.409^#^
Insufficient bone consolidation^b^	-	-	14/49 (29%)	
Heterotopic ossifications	-	16/53 (30%)	-	
Laboratory findings at admission				
Median serum C-reactive protein in mg/l	7.5 (IQR 2.4–32.2)	10.0 (IQR 4.1–32.2)	5.4 (IQR 1.5–34.1)	0.070*
Median blood white cell count in G/l	7.9 (IQR 6.4–9.4)	8.2 (IQR 6.4–9.1)	7.6 (IQR 6.6–10.5)	0.412*

**NOTE**. Data are no. (%) of patients, if not indicated otherwise. P values were calculated between the PJI group and the FDAI group using Mann-Whitney U test (*) or Fisher’s exact test (^#^). IQR, interquartile range.

^a^ Including swelling, erythema, warmth at the index joint site.

^b^ Including delayed union (between 4 and 6 months) and non-union (after >6 months).

### Performance of diagnostic tests

**Fig 1:**
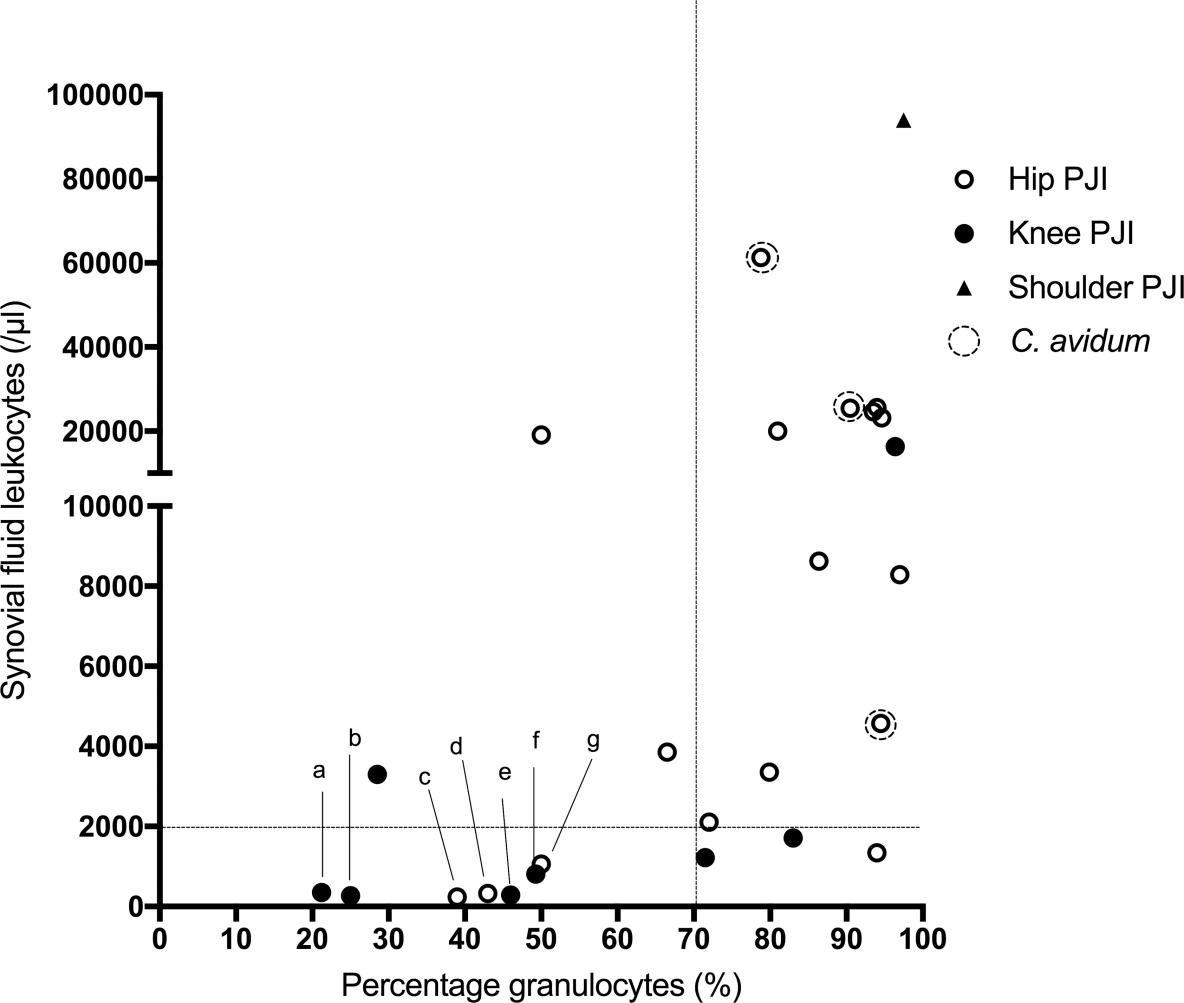
Leukocyte count and granulocytes percentage in 26 PJI patients with complete synovial fluid analysis. The values of three patients were not depicted since the percentage of granulocytes was missing (only the leukocyte count was available). The dotted lines indicate the cutoff values for PJI definition. Seven cases with normal leukocyte count are labeled as »a« through »g« (see details in Table 4).

A summary of non-microbiological and microbiological tests is shown in Table 3. The C-reactive protein (CRP) was increased (>10 mg/l) in 50 of 108 patients (46%), blood white cell count was increased (>10 G/l) in 23 of 106 patients (22%). Whereas CRP was elevated in all infections caused by *C*. *avidum*, in those caused by *C*. *acnes* only 35 out of 76 patients (46%) had a value >10 mg/l (p < 0.001 (Fisher’s exact test)). There was no significant difference of median CRP between different joints in the PJI group. In patients with IAI, histopathology of peri-implant tissue showed inflammation in 47 of 74 patients (64%) with a 100% positivity rate in the subgroup of IAI caused by *C*. *avidum*. In patients with PJI, synovial fluid leukocyte count or granulocyte percentage was increased in 22 of 30 patients (73%). Fig 1 shows the distribution of the synovial fluid leukocyte count in patients with PJI affecting different joints, in whom the leukocyte count was determined. In 7 patients with microbiologically and/or histologically proven PJI, the leukocyte count was normal (dots labeled a through g in Fig 1, details showed in Table 4). Median synovial leukocyte count was higher in hip PJI cases compared to knee PJI cases (8290 leukocytes/μl vs. 1219 leukocytes/μl; p = 0.077). Only for one patient with shoulder PJI synovial fluid leukocyte count was available.

**Table 3:** Diagnostic tests for orthopedic implant-associated infections.

Positive test	All patients (n = 121)	Patients with PJI (n = 62)	Patients with FDAI (n = 59)	P value
**Non-microbiological tests**				
Increased serum C-reactive protein concentration (>10 mg/l)	50/108 (46%)	30/60 (50%)	20/48 (42%)	0.442^#^
Increased blood leukocyte count (>10 G/l)	23/106 (22%)	8/59 (14%)	15/47 (32%)	0.032^#^
Increased synovial fluid leukocyte count or granulocyte percentage^a^	-	22/30 (73%)	-	
Inflammation in peri-implant tissue histopathology	47/74 (64%)	32/46 (70%)	15/28 (54%)	0.215^#^
**Microbiological tests**				
Body fluid culture^b^	29/52 (56%)	20/41 (49%)	9/11 (82%)	0.086^#^
Peri-implant tissue culture	87/103 (84%)	45/61 (74%)	42/52 (81%)	0.502^#^
Sonication fluid culture	79/94 (84%)	42/52 (81%)	37/42 (88%)	0.404^#^

**NOTE**. Data are no. (%) of patients. The percentages were rounded and may not sum 100%. Where the denominator is shown, percentage was calculated for the subgroup in which the test was performed. P values were calculated between the PJI group and the FDAI group using Fisher’s exact test (^#^).

^a^ Defined as synovial fluid leukocyte count >2000 leukocytes/μl or percentage of granulocytes >70%.

^b^ Synovial fluid (in case of PJI) resp. intraoperatively collected peri-implant fluid (in case of FDAI)

**Table 4:** PJI with negative leukocyte count in synovial fluid (see Fig 1). CRP, C-reactive protein; PMN, polymorphonuclear cells (granulocytes); NA, not available.

ID	Gender,Age	Joint	CRP (mg/l)	X-ray	Microbiology (positive specimen)	Pathogen	Pathology	Leukocyte count (/μl)	PMN (%)	Sinus tract	temporal appearance (months)
a	F, 72	Hip	5,6	Loosening	Sonication, tissue samples (1/2)	*P*. *acnes*	Negative	237	39	no	30
b	F, 74	Knee	2,5	Loosening	Sonication	*P*. *acnes*	Negative	273	25	no	32
c	F, 73	Knee	0,6	Loosening	Tissue samples (2/5)	*P*. *acnes*	Negative	287	46	no	25
d	F, 76	Hip	44,4	Stable	Sonication	*P*. *acnes*	Positive	328	43	no	98
e	F, 51	Knee	14,62	Loosening	Synovial fluid	*P*. *acnes*	NA	347	21	no	11
f	F, 79	Knee	9,3	Loosening, ossifications	Synovial fluid	*P*. *acnes*	Positive	813	49	no	NA
g	M, 71	Hip	0,7	Loosening	Tissue samples 2/5, sonication	*P*. *acnes*	Negative	1059	50	no	324

**Fig 2:**
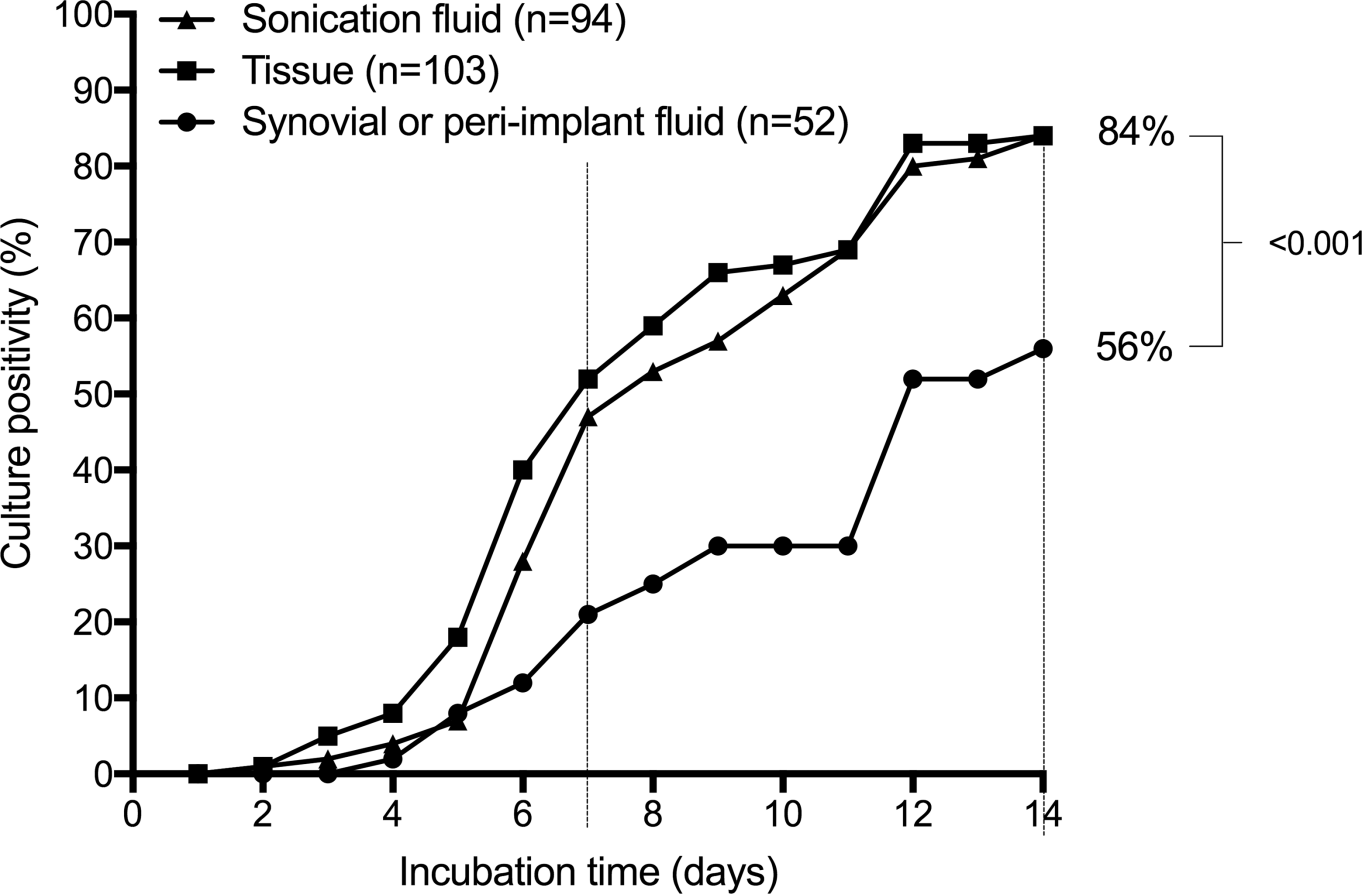
Times to culture positivity of synovial or peri-implant fluid, peri-implant tissue and sonication fluid. The dotted lines indicate the incubation time of 7 and 14 days.

Among microbiological tests, culture of sonication fluid of the explanted orthopedic implant and of periimplant tissue showed a high detection rate of *Cutibacterium* (both 84%), whereas synovial or peri-implant fluid culture was significantly inferior regarding sensitivity (54%). Times to culture positivity of synovial fluid, peri-implant tissue and sonication fluid are shown in Fig 2. After 7 days of incubation the positivity rates were 21%, 53% and 47%, respectively.

### Microbiological findings

Among 121 orthopedic IAI, 109 (90%) were caused by *C*. *acnes* and 12 (10%) by *C*. *avidum*, the latter included seven hip PJI and five FDAI involving humeral (n = 3) and femoral (n = 1) fixation plate and one anchorage in the shoulder joint. In 13 patients (11%, 10 with PJI and 3 with FDAI), co-infection with other pathogen(s) was found, including coagulase-negative staphylococci (n = 11), *Granulicatella adiacens* (n = 1), *Finegolida magna* (n = 1), *Enterococcus faecalis* (n = 1) and *Parvimonas micra* (n = 1). Two patients had a mixed infection with more than two pathogens.

**Fig 3:**
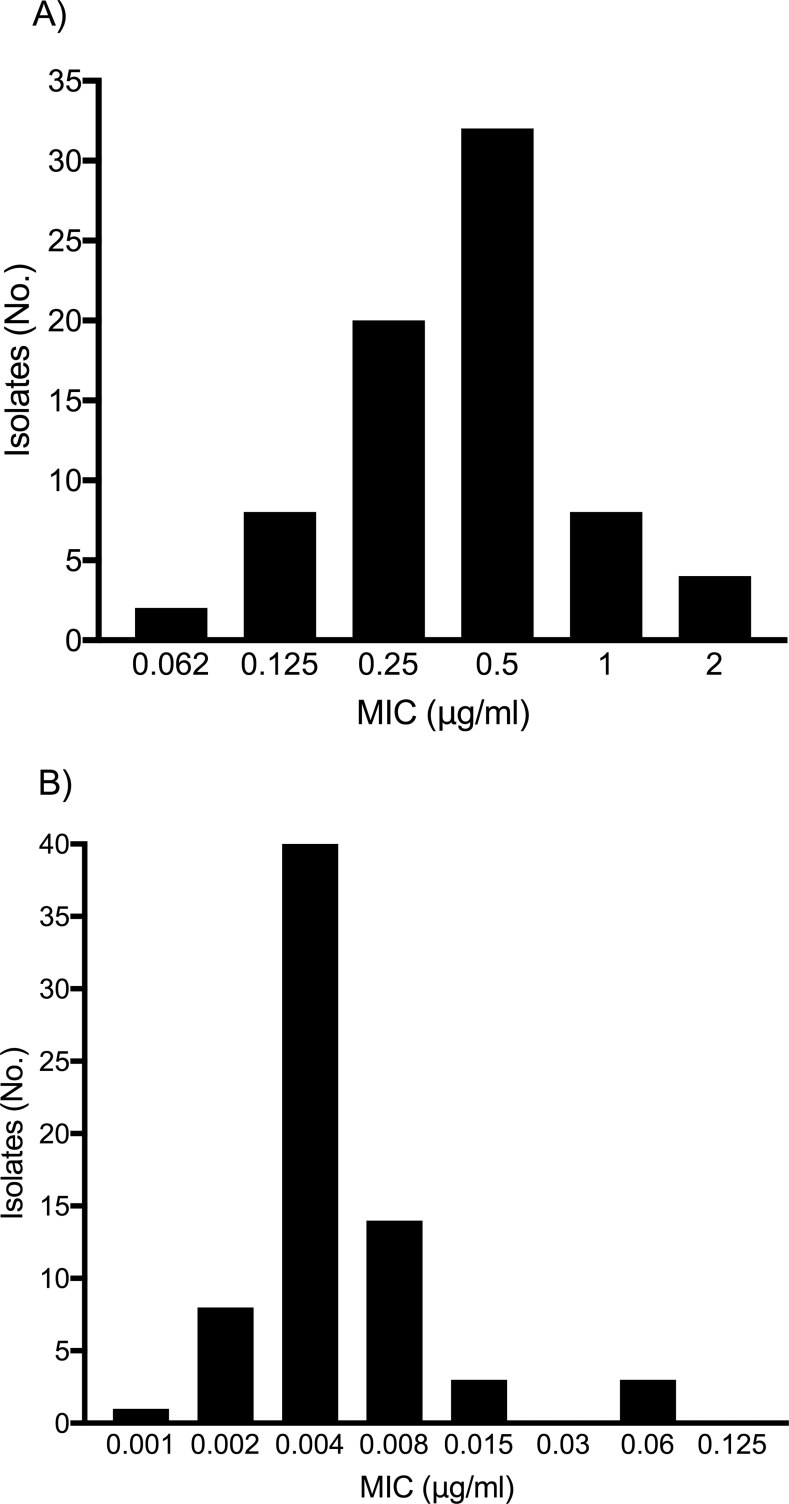
Susceptibility of *Cutibacterium* spp. to levofloxacin (A) and rifampin (B), expressed as distribution of MIC values. MIC, minimal inhibitory concentration.

The distribution of MIC (minimal inhibitory concentration) values is presented in Fig 3. The majority of tested *Cutibacterium* strains had MIC values <2 μg/ml for levofloxacin (37 isolates) and MIC values <0.125 μg/ml for rifampin (32 isolates), with similar distribution in *C*. *acnes* and other *Cutibacterium* spp.. There are no established breakpoints for *Cutibacterium* spp., but authors suggested for levofloxacin low-level resistance at MIC between 0.5 μg/ml and 6 μg/ml and high-level resistance at MIC >6 μg/ml [31]. No resistance to clindamycin or amoxicillin was observed.

### Surgical treatment

In PJI, two-stage exchange of the prosthesis was performed in 37 patients (60%), most commonly using a long interval of ≥6 weeks (30 of 37 patients), one-stage exchange was performed in 15 patients (24%), including six patients with only partial prosthesis exchange of the loose component due to preoperatively presumed aseptic failure. Prosthesis retention was performed in 5 patients (8%), 4 prostheses were permanently removed and one patient was treated conservatively due to impaired general condition and high surgical risk. FDAI were predominantly treated with one-stage exchange (n = 29, 49%), followed by permanent removal of the fixation device (n = 12, 25%) as sufficient bone consolidation was achieved. The remaining cases were treated with two-stage exchange (n = 10, 17%) and retention of the implant (n = 8, 14%), predominantly in acute infections presenting less than 6 weeks after implantation.

### Antimicrobial treatment

The majority of patients (84 of 113 patients, 74%) was treated with a rifampin combination (combined with either amoxicillin or levofloxacin) aiming at eradicating the implant-associated infection, whereas 23 patients (20%) received a suppression treatment with amoxicillin or clindamycin and 6 patients (5%) received no antimicrobial treatment.

## Discussion

Previous reports on *Cutibacterium* IAI predominantly described shoulder PJI [12–15]. In this cohort, we report a high proportion of infection located on lower extremities, mainly hip PJI. Similarly to other reports [8, 13, 15, 16, 32], there was a predominance of males (70% of patients in our cohort), probably reflecting the different distribution of body hair between sexes. Most *Cutibacterium* infections (82%) occurred late after implantation, mostly classified as delayed or late infections, as previously reported [9, 14]. Despite adequate pre-operative skin antisepsis procedures, perioperative contamination occurs as *Cutibacterium* spp. usually reside in the sebaceous glands located in dermal skin layers [33]. As hematogenous spread by this anaerobic pathogen is extremely rare, a long-term silent colonization of the implant before evoking inflammatory changes in the tissue must be assumed.

Slow growth, low microbial burden colonizing the implant, anaerobic growth requirement and low virulence of *Cutibacterium* are delaying the clinical manifestations of IAI. Importantly, persistent or increasing pain at joint site was present in the majority of patients (80%), whereas local signs of inflammation were reported only in 28%. In contrast to PJI, which mostly manifested late, approximately one third of FDAI manifested within the first three months after surgery. This difference may be explained by less soft tissue around the fixation device compared to the joint prosthesis, making local signs of inflammation earlier visible. Unspecific or subtle clinical signs and symptoms of infection evoked by *Cutibacterium* may suggest an aseptic etiology of the implant failure, but does not exclude a low-grade infection.

The performance of conventional preoperative and intraoperative diagnostic tests in our cohort was low, contributing to late diagnosis of IAI. In particular, laboratory parameters in serum and blood were normal in the majority of patients, as reported by others [13, 32, 34, 35]. Joint aspiration with determination of leukocyte count and microbiological analysis is the cornerstone in the preoperative evaluation of a painful prosthetic joint. However, low positivity rate of synovial fluid culture (56%) and leukocyte count (73%) was observed, as reported by others [13, 32, 35]. Interestingly, also histopathological results showed inflammation indicating infection in only 64%.

The low microbiological yield may be explained by the strong ability of *Cutibacterium* to adhere to the implant surface and its change from the planktonic to the biofilm phenotype. Prolonged culture incubation improved the diagnosis of *Cutibacterium* IAI. Only approximately one fifth (synovial or peri-implant fluid) and one half (sonication fluid) of specimens grew *Cutibacterium* within the first 7 days of incubation. These findings support the need for incubation period of 14 days in both aerobic and anaerobic culture media, as previously proposed [36]. Other authors highlighted an even longer incubation time of 21 days [37], however this prolongation holds the increased risk of contamination. Non-microbiological findings may support the clinical suspicion of *Cutibacterium* orthopedic IAI, in spite of normal laboratory and negative microbiological tests, including radiological features such as early loosening, heterotopic ossifications or insufficient bone consolidation.

Within the *Cutibacterium* genus, *C*. *acnes* are most common. However, other species were infrequently described, including *P*. *avidum*, *P*. *granulosoum*, *P*. *lymphophilum* and *P*. *propionicum* [2]. Data on clinical characteristics of these non-*C*. *acnes* isolates are limited to case reports. Whereas *C*. *acnes* were usually described in IAI, *C*. *avidum* caused also infections in absence of foreign bodies such as splenic or perianal abscess [38] and infections after breast surgery without implant [39–41]. Interestingly, in our cohort we found twelve IAI caused by *C*. *avidum*, of whom seven involved a hip prosthesis. In contrast to *C*. *acnes* which is commonly found in oily, sebum-rich areas, *C*. *avidum* is found only in areas rich with sweat glands, namely anterior nares, axilla, rectum and due to spread from rectum, in groin [42]. Therefore it is not surprising that majority of *C*. *avidum* infections in our cohort occurred after hip replacement, as recently shown in several reports focusing on infections after hip arthroplasty [43–45]. In line with previous reports, we noted significantly higher positivity rate of diagnostic tests for infections caused by C. avidum with 100% for elevated CRP, synovial fluid leukocyte count and periprosthetic tissue histopathology. These findings reflect the higher virulence of this specific *Cutibacterium* species as described in earlier reports.

Eradication of IAI is best achieved by a combination of both appropriate antimicrobial and surgical treatment. Due to its broad antimicrobial susceptibility (including to rifampin), the majority of *Cutibacterium* orthopedic IAI can be theoretically treated with one-stage revision, providing that the surrounding soft tissue is not compromised and all foreign material and dead tissue can be removed during debridement [24]. The activity of rifampin against *C*. *acnes* biofilms was demonstrated in vitro and in an experimental model of foreign-body infection [46, 47]. A combination regimen with rifampin was subsequently integrated in treatment recommendations. The aberrant use of antibiotics in acne may lead to the development of *C*. *acnes* strains with cross-resistance to various antibiotics with clinical impact in all diseases caused by *Cutibacterium* species [48]. Despite MIC values for rifampin and levofloxacin in *Cutibacterium* are generally in the susceptible range, emergence of resistance to both antibiotics has been reported [31, 49] and antimicrobial susceptibility testing is essential. As a dramatic decrease of clindamycin serum concentrations was shown in patients with staphylococcal osteoarticular infections treated with oral clindamycin-rifampicin combination, it is considered a second line combination partner for antibiotic regimens with rifampicin aiming at infection eradication, unless clindamycin serum concentration is thoroughly controlled [50].

In conclusion, due to heterogeneous, subtle and atypical clinical presentation, *Cutibacterium* IAI is often diagnosed late in the disease course. Conventional microbiological tests showed limited sensitivity, which can be improved by prolonged culture incubation and implant sonication. Due to lack of reliable diagnostic tests for low-grade IAI, some aseptic conditions may be misdiagnosed as infections and vice versa. With additional knowledge and better diagnostic tests, *Cutibacterium* infections are expected to be more often reliably diagnosed or excluded in future, improving the long-term treatment outcome.
